# Mr. Aberdour, on Strictures of the Urethra

**Published:** 1800-11

**Authors:** Alexander Aberdour

**Affiliations:** Prince's Place, Lambeth


### *To the Editors of the Medical and Phyſical Journal.*

GENTLEMEN,

IT afforded me no ſmall degree of ſatisfaction to obſerve in your Fourteenth Number, Mr. Carliſle's Remarks on the Uſe of Cauſtic, ſeveral of which appear to me to be pertinent and judicious; his motives every one muſt approve.

Induced by the ſame ſentiments, I beg leave to contribute my mite through the channel of your uſeful Journal.

Mr. Home, in his Treatiſe on Strictures, ſpeaking of the Urethra, obſerves, that "When the urine paſſes, the canal is large; large; when the femen is thrown out it is fmall, the membrane having a power of adapting itfelf to thefe two different ftates; and this power of action within itfelf is fimilar to mufcular contraction and relaxation."

Does this action in the canal of the urethra take place in reality? I apprehend not; and the matter may be explained in another way.

In the emiffion of femen, the penis is commonly erected; a greater quantity of blood than ufual is at this time lodged in the cells of the corpora cavernofa penis, which *compreffes* the fides of the urethra; that this happens I infer from the difficulty that occurs during erection to void urine, which paffes then in a fmall divided ftream.

We judge of a ftricture in the urethra by the difficulty the patient complains of in making water; by his frequent defire to void it, in doing which it is attended with pain, and comes away in fmall quantities, either in drops or in a narrow contracted ftream; and laftly, by examination with a bougie or catheter.

A difcharge from the urethra, fimilar to a gleet, is I prefume not an unfrequent attendant on ftricture; and extraneous fubftances, fuch as ftones, are fometimes found in the paffage, which, however, are readily diftinguifhed by the touch on introducing the found.

With refpect to the proximate caufe, we learn from Mr. Home, who has examined the difeafe by diffection after death, that there is a doubling of the membrane and narrowing of the urethra in particular parts, which when the contraction is increafed, becomes a ridge projecting into the canal, behind which ulceration is fometimes obferved.

In the treatment of ftrictures, without having recourfe to the knife, two methods have been adopted; the one is by the introduction of bougies, and the other by the application of cauftic.

In the former, the bougies act by their mechanical preffure, and thus dilate the canal of the urethra; this dilatation is however merely temporary, and I have known gentlemen ufe bougies for years without the ftricture being removed. The other mode of treatment, viz. the application of cauftic, was recommended and practifed by the late Mr. Hunter; fince his death, it has been greatly improved by Mr. Home, whofe knowledge and great experience in the treatment of ftrictures every one will allow.

This practice of applying cauftic to ftrictures of the urethra is ftill in its infancy, and it becomes an object of importance to know whether or not it anfwers the end propofed; at the fame time time I beg leave to ſay that it has aſtoniſhed me to obſerve gentlemen condemn the practice without giving it a trial.

Mr. Carliſle has mentioned a caſe where the cauſtic was uſed, and followed by a profuſe hæmorrhage.

It would be reflecting on his practice, to ſuppoſe in the caſe he alludes to, the cauſtic had been too long or too extenſively applied, by which a high degree of inflammation might have been induced, and thus have occaſioned the hæmorrhage. The particulars of the caſe I know not; but in a caſe of highly inflamed phymoſis, I have known the application of half a dozen of leeches to the prepuce followed by a profuſe bleeding, which was, however, attended with the beſt effects.

Before uſing the cauſtic, it is neceſſary to aſcertain the diſeaſe, with its ſituation; and this we do by means of a bougie, made either of poliſhed leather or the elaſtic gum; the latter is preferable, as it is not ſo hard, and is ſufficiently elaſtic. On introducing it into the urethra, we are not to imagine, that in every ſtoppage we meet with, there is a ſtricture, becauſe the point of the bougie may have plunged into one of the lacunæ; add to this, the effect induced by irritation. We are to pauſe ſome little time, then to turn round the bougie, and raiſing the point by lowering the other end, we try to puſh it gently on.

Let us next ſuppoſe that we meet with an obſtruction, and that the caput gallinaginis (which is commonly ſituated in an adult little more than eight inches from the external orifice of the urethra) is out of the queſtion, we are now to withdraw the bougie, and introduce another made of ſoft materials, exactly to the ſame extent, by which we take off the impreſſion; with this and the other ſymptoms, we judge of the ſtricture. This being determined, we are then to introduce a third bougie, properly armed with cauſtic, and oppoſe it to the ſtricture for the ſpace of fifty ſeconds, or a minute; beyond ninety ſeconds I have not applied a cauſtic bougie to a ſtricture.

With reſpect to the ſize of bougies, I confeſs that I have not been in the practice of uſing ſuch large ones as others have done; thoſe that I have employed, have not exceeded in diameter a fifth, or at moſt a quarter of an inch.

The caſts of the urethra taken by Mr. Home from the dead ſubject, cannot regulate the urethra of a living perſon. He talks of the canal, meaning the urethra, paſſing over the proſtate gland. Anatomy tells me, that the portion of the urethra which is next to the bladder, is ſurrounded by the proſtate gland; of courſe, it paſſes *through*, and not *over* the gland.

With regard to the effects of cauſtic, although I cannot in my ſhort experience boaſt of cures performed, yet I have noticed, after the uſe of cauſtic, that the urine has been voided in a free regular ſtream; that the pain attending it is trifling; and no one untoward ſymptom has hitherto occurred.

I was not a little ſurpriſed in the caſe of an officer belonging to the navy, where I had removed two ſtrictures; the firſt, four inches and a half from the external orifice, ſeemed to be removed by two touches of the cauſtic; the other, ſix inches and a half from the orifice, required twelve applications, in the laſt a ſlight hæmorrhage enſued; a common ſized bougie paſſed eaſily into the bladder. I expected now to have no difficulty; two days after, however, I was ſurpriſed to find that I could not paſs the bougie beyond the place where the firſt ſtricture had been. I had recourſe again to the cauſtic, which was only uſed once; no difficulty has ſince occurred in paſſing a bougie on to the bladder, and the gentleman is now without complaint. The diſeaſe was of two years ſtanding.

I have the honour to be, Gentlemen,

Your moſt obedient humble ſervant,

ALEXANDER ABERDOUR.

*Prince's Place, Lambeth,*

*Oct.* 13, 1800.

